# Relationship between nonperfusion area from widefield optical coherence tomography angiography and macular vascular parameters in diabetic retinopathy

**DOI:** 10.1007/s10792-023-02882-0

**Published:** 2023-10-18

**Authors:** Ami Konno, Akihiro Ishibazawa, Lucas De Pretto, Akito Shimouchi, Tsuneaki Omae, Young-Seok Song

**Affiliations:** 1 Department of Ophthalmology, Asahikawa Medical University, Midorigaoka Higashi 2-1-1-1, Asahikawa, 078-8510 Japan; 2 Nuclear and Energy Research Institute IPEN-CNEN/SP, Sao Paulo, Brazil

**Keywords:** Optical coherence tomography angiography, Diabetic retinopathy, Nonperfusion area, Macular vascular parameters

## Abstract

**Purpose:**

To evaluate the relationship between the nonperfusion area (NPA) from widefield optical coherence tomography angiography (OCTA) and macular vascular parameters in diabetic retinopathy (DR).

**Methods:**

In total, 51 eyes from 51 patients with proliferative DR (PDR) or moderate/severe non-PDR were included. Widefield OCTA using the Zeiss Plex Elite 9000 was performed. A semi-automatic algorithm calculated the percentages of the NPA within the total image. Macular OCTA (3 × 3 mm and 6 × 6 mm area) was scanned using the RTVue-XR Avanti. Vessel density (VD) was automatically separated into the superficial (SCP) and deep capillary plexus (DCP), and foveal avascular zone (FAZ) measurements were computed according to the parafoveal (1–3 mm) and perifoveal (3–6 mm) regions.

**Results:**

A negative correlation was found between the average VD of the SCP and DCP obtained 3 × 3 mm and 6 × 6 mm area and the NPA. Multiple regression analysis revealed that the temporal–perifoveal region most negatively correlated with the NPA (*r* =  − 0.55, *p* < 0.0001). No correlation was found between FAZ measurements and DR severity (area, *p* = 0.07; perimeter, *p* = 0.13).

**Conclusion:**

Diabetic macular nonperfusion was significantly associated with the NPA from widefield OCTA. In particular, the temporal–perifoveal DCP disorder may be a sensitive indicator of wide NPA.

## Introduction

The leading microvascular ocular complication of diabetes is a diabetic retinopathy (DR), a serious disease that causes blindness in active age [1, 2]. Fluorescein angiography (FA) is important and clinically useful examination to detect the vascular perfusion and the nonperfusion area (NPA) or retinal neovascularization (NV) in the retina. However, FA is invasive and can cause complications, which are severe life-threatening reactions. They are extremely rare but may occur [3]. Thus, FA is unrepeatable on the same day or cannot be performed frequently. OCTA is a noninvasive and useful tool for detecting diabetic vascular disorders, such as the NPA, intraretinal microvascular abnormalities, and NV, as we described previously [4–6]. Since fluorescence leakage does not affect OCTA image, which enables us to measure the NPA more accurately than FA images [7]. It is reported that the percentage of the NPA quantified using widefield (12 mm × 12 mm) OCTA images showed a statistically significant increase as DR worsened [8]. We also investigated whether the NPAs are distributed arterial-adjacent or venous-adjacent in DR using widefield OCTA. Smaller NPA tended to be arterial-adjacent, whereas larger nonperfusion tended toward veins [9]. Although widefield OCTA contributes greatly to the clinical evaluation of DR, only limited devices can be used for scanning, which may fail mainly because of the long scanning time and poor fixation of the patients. In contrast, OCTA scans centered on the fovea can be taken in a short time more successfully than widefield ones. If macular nonperfusion can reflect peripheral ischemia, it may bring substantial benefits for usual clinical practice in evaluating DR severity. In this study, we investigated the relationship between the NPA from widefield OCTA and vascular changes from macular OCTA in eyes with relatively severe DR stages.

## Methods

### Study population

Asahikawa Medical University approved the protocol for this study. The study complied with the Declaration of Helsinki. Patients with diabetes mellitus (DM) were retrospectively recruited from the Asahikawa Medical University between February 2019 and May 2020. Patients had undergone widefield OCTA, 3 × 3 mm and 6 × 6 mm OCTA scans centered on the fovea at the first visit. These patients underwent a comprehensive ophthalmic examination. Structural OCT findings determined the presence of diabetic macular edema (DME). We included the eyes with the same stage and moderate non-proliferative DR (NPDR) or worse in this study, retina specialists classified DR according to the International Clinical Diabetic Retinopathy Severity Scale [10]. We excluded the eyes with mild NPDR from the analysis in this study. This is because these eyes may have capillary dropout, but these areas were below the detection threshold of our algorithm. The exclusion criteria were the following; the presence of media opacities such as severe cataract or vitreous hemorrhage, a history of vitrectomy, other choroidal diseases such as age-related macular degeneration, retinal artery or vein occlusion, glaucoma, prior anti-vascular endothelial growth factor (VEGF) treatments, and/or panretinal photocoagulation (PRP) within 6 months. In subjects whose eyes met the inclusion/exclusion criteria, one eye was randomly selected for imaging.

### Macular OCTA

**Fig. 1:**
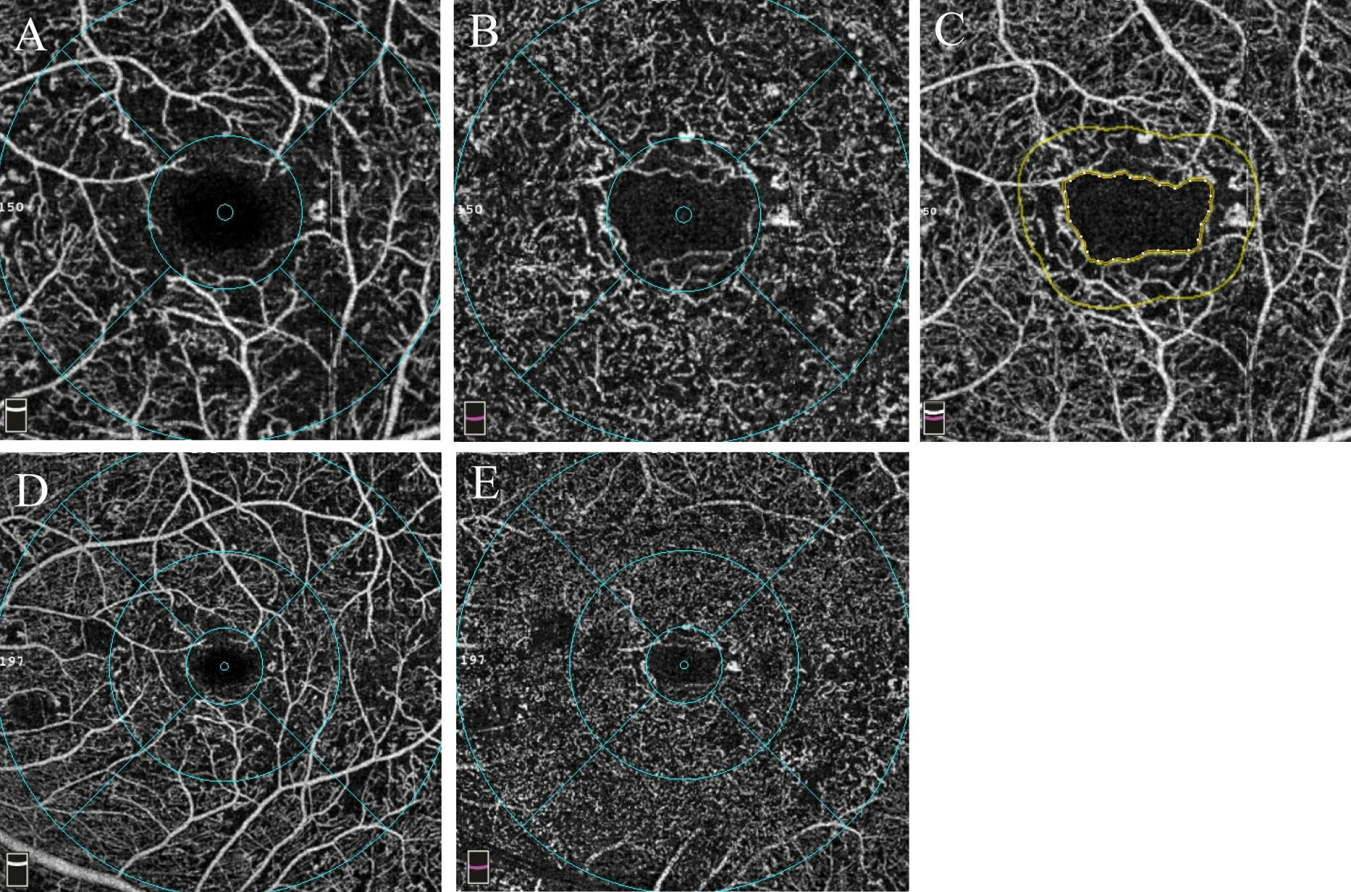
OCTA images, the upper row is 3 × 3 mm OCTA and the bottom row is 6 × 6 mm OCTA. The grid consists of two or three concentric circles at the fovea with diameters of 1, 3, and 6 mm based on the Early Treatment of Diabetic Retinopathy Study. The vessel density (VD) is calculated with the annulus automatically centered at the level of the superficial capillary plexus (SCP) (**A**, **D**) and deep capillary plexus (DCP) (**B**, **E**). **C** Foveal avascular zone (FAZ) measurements are area and perimetry

Patients were imaged using a commercially available spectral-domain OCT (RTVue-XR Avanti; Optovue, Inc., Fremont, CA, USA) with split-spectrum, amplitude-decorrelation angiography software. This device has a 70,000 A-scans/second and uses a light source centered at 840 nm and a bandwidth of 45 nm. Two consecutive B-scans, 3 × 3 mm macular region containing 304 A-scans and 6 × 6 mm containing 400 A-scans, were captured at each sampling location. For each patient, we obtained 3 × 3 mm and 6 × 6 mm scans centered on the fovea. The built-in AngioVue software (version 2018.0.0.18) automatically segmented En-face OCT angiograms. The superficial capillary plexus (SCP) was located between the inner limiting membrane (ILM) and 9 µm above the junction between the inner plexiform layer and the inner nuclear layer (IPL–INL); whereas, the deep capillary plexus (DCP) was located between 9 µm above the IPL–INL junction and 9 µm below the outer plexiform layer and the outer nuclear layer (OPL–ONL) junction. The macular map was defined on the basis of the Early Treatment of Diabetic Retinopathy Study. The grid consisted of three concentric circles at the fovea with diameters of 1, 3, and 6 mm. The parafovea was considered as the area between the inner (1 mm) and middle (3 mm) rings (Fig. 1A, B) and the area between the middle (3 mm) and outer (6 mm) rings (Fig. 1D, E) defined as the perifovea. The vessel density (VD) of the SCP and DCP was automatically generated and recorded by the device. The foveal avascular zone (FAZ) area was also evaluated. This was automatically measured from the full retinal thickness and generated by automatically segmenting the ILM to 9 µm below the outer plexiform layer (Fig. 1C). We only adopted OCTA images with a signal strength greater than 6/10 in this study.

### Widefield OCTA

**Fig. 2:**
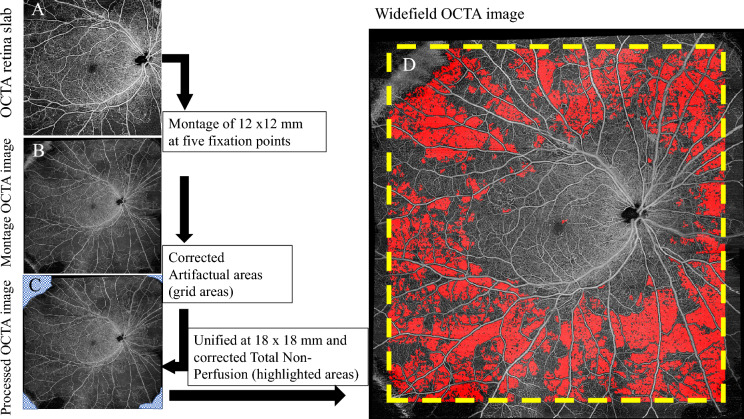
Workflow used in detecting and demarcating capillary nonperfused areas. **A** 12 × 12 mm OCTA centered at the fovea of the full retina slab. **B** Montage image generated from five OCTA images (each 12 × 12 mm) at five fixation points. **C** OCTA image manually traced regions with artifacts (grid areas). **D** Corresponding the widefield OCTA image with the algorithm’s results overlaid. Widefield OCTA is unified at 18 × 18 mm by a yellow dot-line. The red -highlighted areas show the total percentage of the nonperfusion area (TNPA). TNPA from the representative example shows 30.0% in the right eye with PDR (58-year-old male)

Patients were imaged using a Plex Elite 9000 device (Carl Zeiss Meditec, Inc., Dublin, CA, USA). The instrument performed 100,000 A-scans/second and used light sources centered between 1040 and 1060 nm. At each sampling position, two consecutive B-scans of 12 × 12 mm were taken, each containing 500 A-scans (Fig. 2A). A montage image, which consisted of five OCTA volumes (each 12 × 12 mm) at different fixation points: central, superior nasal, inferior nasal, inferior temporal, and superior temporal, was generated using the instrument’s built-in software (Fig. 2B). En -face OCTA images of the full retinal thickness were used for analysis, with the ILM automatically segmented to 70 µm above the retinal pigment epithelium.

### Identification and quantification of capillary NPAs

Custom software written in MATLAB (MathWorks, Natick, MA, USA) was used to semi-automatically detect the NPAs in OCTA images. This software has been fully described in a previous work; Alibhai et al. discovered this algorithm [8]. Briefly, the software uses a combination of spatial variance metrics and flooding-based algorithms to find regions of possible nonperfusion. In addition to excluding artifactual regions prior to analysis, users can also manually modify (i.e., exclude and/or add) identified segments to reduce potential artifacts (Fig. 2C). Because the image quality in the periphery is not guaranteed due to segmentation errors, widefield OCTA was standardized at 18 × 18 mm (Fig. 2D). The scan density used in widefield montages is too low to resolve individual capillaries in small areas, such as those surrounding the focal avascular zone. Therefore, they defined a minimum area threshold to be selected by the algorithm with the goal of improving performance reliability. Therefore, for automatic identification, nonvascular contiguous areas consisting of ≧250 pixels (approximately 0.15 mm^2^) were defined as NPA; the FAZ and optic nerve head were automatically detected and were manually excluded. The percentage of capillary nonperfusion area to the total area was calculated as follows:

Total percentage of nonperfusion area (TNPA) = (sum of nonperfusion area/ (field-of-view area–excluded area) × 100%) (Fig. 2D).

### Statistical analysis

Categorical data were presented as the number and percentages and analyzed using the chi-squared (*χ*2) test. Nonparametric categorical data were analyzed using the Mann–Whitney test. Quantitative data were tested for normality using the Shapiro–Wilk test, assuming normality at *P* > 0.05. Normally distributed variables were expressed as mean ± standard deviation (SD) and analyzed by one-way analysis of variance (ANOVA) for three independent groups; whereas, nonparametric data were presented as median and interquartile range and analyzed by the Kruskal–Wallis (KW) test. Significant KW findings were followed by post hoc multiple comparisons using Bonferroni-adjusted tests to detect significant pairs. The Spearman correlation coefficient (rho) was used to evaluate nonparametric correlations. Finally, multiple regression analysis was used to assess which part of the vessel density (VD) would have the strongest relationship with the TNPA.

For all tests, values of *P* < 0.05 were considered statistically significant, and *P* < 0.001 was considered highly significant. Prism 7 (GraphPad Software, Inc., La Jolla, CA, USA) and IBM SPSS Statistics for Windows version 25 (IBM Corp., Armonk, NY, USA) were used for statistical analysis.

## Results

### Baseline characteristics

A total of 131 patients were enrolled in this study. A total of 57 patients were excluded due to the presence of concomitant ocular pathology, treated with VEGF injections and/or PRP within 6 months. We further excluded 23 eyes due to poor OCTA image quality resulting from excessive motion artifacts and poor OCT signal penetration. A total of 51 eyes from 51 participants (33 men and 18 women) were identified. The median age of these participants was 58 years. Of the 51 eyes, 14 were clinically determined as moderate NPDR, 12 as severe NPDR, and 25 as PDR. Table 1 shows the ocular parameters and characteristics of the included eyes. There were no significant differences among the groups with respect to best-corrected visual acuity (BCVA), intraocular pressure, central macular thickness, or history of anti-VEGF treatment. In addition, 6 (50%) eyes with severe NPDR and 23 (92%) with PDR had been treated with PRP.

**Table 1:** Comparison between the studied groups regarding basic characteristics

Variables	Moderate NPDR, *n* = 14	Severe NPDR, *n* = 12	PDR, *n* = 25	*P* value
Age	62.9 ± 10.5	56.8 ± 12.1	55.3 ± 12.0	0.149*
Gender, *n*				0.832^†^
Male	10	8	19	
Female	4	4	6	
Log MAR Visual acuity	0 ± 0.10	0.05 ± 0.13	0.09 ± 0.13	0.07*
Intraocular pressure, mmHg	15.8 ± 1.3	14.2 ± 4.2	15.6 ± 2.4	0.283*
Central macular thickness, μm	291.7 ± 50.7	285.1 ± 78.3	306.2 ± 79.5	0.686*
Anti-VEGF injection, *n* (%)	1 (7%)	1 (8%)	3 (12%)	0.873^‡^
PRP received, *n* (%)	–	6 (50%)	23 (92%)	0.008^§^

All values are given as mean ± standard deviation

*NPDR* Non-proliferative Diabetic Retinopathy, *PDR* Proliferative Diabetic Retinopathy, Log *MAR* Logarithm of the Minimum Angle of Resolution, *VEGF* Vascular Endothelial Growth Factor, and *PRP* Panretinal Photocoagulation

*ANOVA

^†^χ^2^ test

^‡^Kruskal–Wallis test

^§^Mann–Whitney test

### Relationship between macular vascular parameters and DR severity

VD of the SCP and DCP of the 3 × 3 mm OCTA showed the same trends. Both decreased with increasing DR severity, with significant differences only between the moderate NPDR and PDR groups (SCP, *p* = 0.049; DCP, *p* = 0.009) (Table 2). We had the same result as in the case in the 6 × 6 mm OCTA (SCP, *p* = 0.02; DCP, *p* = 0.009) (Table 2). There was no correlation between FAZ measurements and DR **s**everity (area, *p* = 0.07; perimeter, *p* = 0.13) (Table 2).

**Table 2:** Relationship between OCTA parameters and severity of diabetic retinopathy

Variable		Moderate NPDR, *n* = 14	Severe NPDR, *n* = 12	PDR, *n* = 25	*P* value
	Mean ± SD				
TNPA (%)		9.7 ± 3.9	17.4 ± 6.0	23.9 ± 8.9	< 0.0001*
3 mm Whole (%)					
Superficial		41.6 ± 2.9	38.2 ± 4.0	37.7 ± 5.8	0.049*
Deep		44.0 ± 3.2	44.3 ± 3.9	39.9 ± 5.6	0.009*
	Median (IQR)				
6 mm Whole (%)					
Superficial		45.0 (44.1, 48.4)	44.3 (40.2, 46.7)	42.1 (37.9, 45.2)	0.02^†^
Deep		46.1 (41.1, 48.7)	43.9 (39.3, 45.8)	40.8 (37.8, 43.2)	0.009^†^
FAZ					
area (mm^2^)		0.3 (0.2, 0.4)	0.3 (0.2, 0.4)	0.3 (0.3, 0.5)	0.07^†^
perimeter (mm)		2.2 (1.9, 2.6)	2.2 (1.8, 2.5)	2.5 (2.1, 3.4)	0.13^†^

Normally, distributed variables are expressed as mean ± standard deviation; while, nonparametric data were presented as median and interquartile range

*OCTA* Optical Coherence Tomography Angiography, *NPDR* Non-proliferative Diabetic Retinopathy, *PDR* Proliferative Diabetic Retinopathy, *TNPA* Total Percentage of Nonperfusion Area, and *FAZ* Foveal Avascular Zone

*ANOVA

^†^Kruskal–Wallis test

### Relationship between the TNPA of widefield OCTA and DR severity

In the montage OCTA images, the median TNPAs were 9.7% in moderate NPDR, 17.4% in severe NPDR, and 23.9% in PDR. A significant difference in the TNPA was found between the moderate NPDR and PDR groups (*p* < 0.0001). The TNPA proportionally increased with DR severity (Table 2).

### Correlations between macular vascular parameters and TNPA

A negative correlation was found between the average VD of the SCP and DCP obtained in the 3 × 3 mm and 6 × 6 mm area and TNPA (Table 3). Of these, the average DCP-VD obtained in the 6 × 6 mm area had the strongest inverse relationship with the TNPA (*r* =  − 0.47, *p* = 0.0005) (Table 3). Comparison by quadrant revealed that the temporal quadrant of both the parafovea and perifovea negatively correlated with the NPA (parafovea, *r* =  − 0.42, *p* = 0.003; perifovea, *r* =  − 0.55, *p* < 0.0001) (Table 3). Stepwise regression analysis demonstrated that the temporal–perifoveal DCP was the best predictive factor for the TNPA (Table 4). The adjusted coefficient of determination was not so high (*R*^2^ = 0.299).

**Table 3:** Correlations between OCTA vascular parameters and TNPA

Parameter	Rho	*P* value	Parameter		Rho	*P* value	Parameter		Rho	*P* value
3 mm			6 mm				6 mm			
SCP-VD			SCP-VD				DCP-VD			
whole	− 0.34	0.014*	whole		− 0.38	0.006*	whole		− 0.47	0.0005^†^
temporal	− 0.29	0.037*	parafoveal	temporal	− 0.32	0.026*	parafoveal	temporal	− 0.42	0.003*
superior	− 0.31	0.027*		superior	− 0.4	0.004 *		superior	− 0.26	0.066
nasal	− 0.18	0.19		nasal	− 0.31	0.028*		nasal	− 0.29	0.04*
inferior	− 0.27	0.054		inferior	− 0.31	0.029*		inferior	− 0.38	0.007*
DCP-VD										
whole	− 0.29	0.039*	perifoveal				perifoveal			
temporal	− 0.22	0.13		temporal	− 0.41	0.003*		temporal	− 0.55	< 0.0001^†^
superior	− 0.15	0.28		superior	− 0.33	0.02*		superior	− 0.44	0.002*
nasal	− 0.3	0.03*		nasal	− 0.32	0.03*		nasal	− 0.25	0.08
inferior	− 0.25	0.078*		inferior	− 0.33	0.02*		inferior	− 0.39	0.006*

Data are presented as percentages unless otherwise indicated

*OCTA* Optical Coherence Tomography Angiography, *SCP-VD* Superficial Capillary Plexus Vessel Density, *DCP-VD* Deep Capillary Plexus Vessel Density, and *TNPA* Total Percentage of Nonperfusion Area

*P < 0.05

^†^*P* < 0.001

**Table 4:** Stepwise multiple linear regression analysis for the predictors of TNPA

	*R*^2^		Adjusted *R*^2^	SEE	F	*P* value
	0.314		0.299	7.947	21.066	< 0.01
variable	Unstandardized Coefficients		Standardized Coefficients	95% CI of B	t	
	B	Std. Error	Beta			
(constant)	67.94	10.772	–	46.257, 89.624	6.307	< 0.01
DCP-VD						
Perifovea-Temporal	− 1.077	0.235	− 0.56	− 1.549, − 0.605	− 4.590	< 0.01

*TNPA* Total Percentage of Nonperfusion Area, *DCP-VD* Deep Capillary Plexus Vessel Density, *R*^*2*^ Regression coefficient, *SEE* Standard Error of Estimate, and *F* F-ratio

### Case presentation

**Fig. 3:**
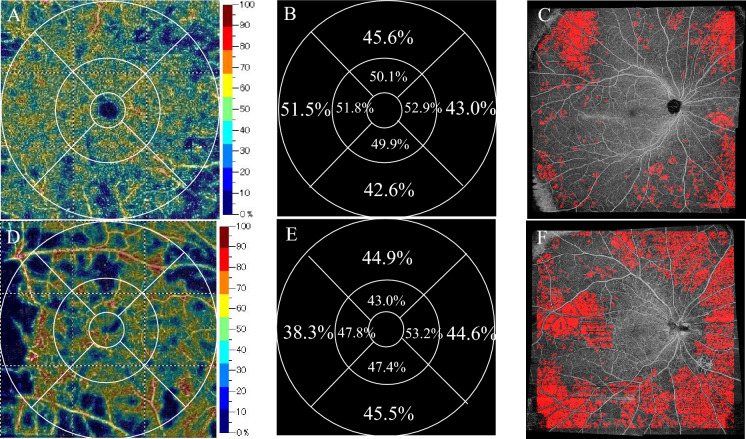
Representative examples showing the total percentage of nonperfusion area (TNPA) with increasing DR severity. The left column shows OCTA images (6 × 6 mm) from DCP: corresponding color-coded vessel density mapping with quantitative data (**A**, **D**). The middle column shows the vessel density (%) of each part of the grid (**B**, **E**). The right column shows the widefield OCTA images of the algorithm performance in detecting capillary nonperfusion (**C**, **F**). The upper row presents data for a 54-year-old man with moderate NPDR, and the bottom row presents that of a 63-year-old woman with PDR. The TNPAs from the widefield OCTA are 11.8% (**C**) and 28.2% (**F**)

The 6 × 6 mm OCTA images from the DCP in the right eye of a 54-year-old man (Fig. 3A) and the right eye of a 63-year-old woman (Fig. 3D) are presented. The former was moderate NPDR, the temporal–perifoveal DCP showed no clear capillary dropout, and the TNPA from widefield OCTA was 11.8% (Fig. 3B, C). In contrast, the latter was PDR, the VD of temporal–perifoveal DCP obviously reduced, and the TNPA from widefield OCTA was 28.2% (Fig. 3E, F).

## Discussion

In this study, we evaluated the relationship between the NPA from widefield OCTA and macular vascular changes in DR. A similar study was performed in the past, but Hajdu et al. assessed peripheral ischemia using ultrawide field angiography (UWFA) [11]. They revealed retinal ischemic index and the amount of micro-aneurysms assessed on UWFA up to peripheral areas are indicators of DR severity but not related to microvascular perfusion status in the SCP and DCP. They used UWFA to assess peripheral retinal ischemia, but are concerned leakage which may obscure ischemic areas. FA is an important imaging, although the leakage of fluorescein and PC scars interfere with the quantification of the NPA. On the other hand, OCTA can clarify the structural details of the NPA and is the same no matter when it is taken. Additionally, widefield OCTA has high sensitivity and specificity for detecting the NPA [12]. This study could quantify peripheral NPA more accurately than previous studies.

Numerous studies have demonstrated that quantitative OCTA metrics on the SCP and DCP correlated with DR severity [13, 14]. In this study, VD of the SCP and DCP also decreased significantly with DR severity (Table 2). Previous studies have suggested that the deep capillary layer was more severely affected in DM [15–17]. The density of the smaller vessels in the deep retinal capillary layer is greater than that in the superficial layer [15, 18]. We hypothesize that capillary dropout occurs more frequently in the deep retinal capillary layer than superficial layer to compensate for the reduced macular blood flow and consequent hypoxia and ischemia during DM. Moreover, many previous studies reported that the FAZ region enlarged with advancing DR severity [19–21]. However, according to Bhanushali et al. and Durbin et al., the FAZ area had the lowest sensitivity and specificity to other vascular parameters investigated as indicators of DR [22, 23]. This finding may be attributed to the high interindividual variability in the FAZ area. This study also showed that the FAZ area and perimeter were not related to DR severity. Therefore, the FAZ parameter may be used as a part of the diagnostic procedure for DR, but may be unsuitable as a parameter when used alone.

Several studies have highlighted the importance of the peripheral retina in DR and found that peripheral retinal ischemia is associated with an increased risk of DR worsening [24–26]. Nicholson et al. emphasized that there was no significant change in the posterior pole; whereas, the difference was predominantly seen in the periphery, with the peripheral NPA being significantly higher in eyes with PDR than in those with NPDR [27]. Peripheral nonperfusion appears to be the determining factor in PDR. We reported that the NPA detected by widefield OCTA increased with worsening DR severity, and only the difference between the moderate NPDR and PDR groups reached significance [9]. This study demonstrated the same result. Cui et al. reported that the detection capability of the NPA from widefield OCTA was comparable with widefield FA images [28]. They supported widefield OCTA as a useful, noninvasive alternative to widefield FA in NPA detection. Consistent with previous reports, the NPA from widefield OCTA correlated with DR severity.

Hirano et al. indicated that 3 × 3 mm macular OCTA images were the best for predicting the presence or absence of DR [29]. However, as retinopathy worsens and, the macular damage aggravates and enlarges, so the 6 × 6 mm macular OCTA images could be more suitable to detect these changes. Specifically, in our study, the average DCP-VD obtained 6 × 6 mm area had the strongest inverse relationship with the NPA. We hypothesize that 3 × 3 mm images to be more appropriate for early DR and 6 × 6 mm images for advanced DR. Alam et al. also observed that the sensitivity of vascular dropout, or VD reduction, is higher in the perifoveal region than in the parafoveal region [30]. The relatively low sensitivity of VD in the parafoveal area could be due to the variable shapes and size of the fovea within 3 × 3 mm images.

A study reported that the capillary dropout in the moderate-to-severe NPDR group mainly occurred in the periphery and most frequently in the temporal quadrant [31]. Pathologic examinations had also shown that vascular abnormalities occur more frequently in the temporal retina than in the nasal retina [32]. Furthermore, a recent study using ultrawide field imaging reported that vascular lesions occurred more frequently in the temporal fields than in the nasal fields [33]. Alam et al. demonstrated that temporal–perifoveal region was the most sensitive region for the early detection of DR [30]. Kaizu et al. reported that capillary dropout associated with DR can be localized with spatial bias by area segmentation measurements using OCTA [14]. The radial peripapillary capillaries (RPC) nourish the inner portion of the nerve fiber layer around the optic disc [34]. The RPC was not depicted in the temporal macula observed with widefield OCTA [35]. Yasukura et al. investigated the NPA of DR in each segmented by the distances from the optic disc on a widefield OCTA image [36]. They revealed that the NPA developed more frequently in the peripheral area from the optic disc and explained this result as follows; the RPC may be associated and the higher perfusion pressure close from the optic disc. We consider that even in the same horizontal raphe, the nasal region is anatomically more likely to be supplied with radial peripapillary capillaries; whereas, the temporal region is more prone to ischemia, and this part must be sensitive for DR progression. In this study, the stepwise multiple linear regression analysis demonstrated that the temporal–perifoveal region of the DCP was the best predictive factor for the NPA. However, the adjusted coefficient of determination was not so high, and it is hard to identify that the NPA can be estimated from only temporal–perifoveal DCP-VD. Ashraf et al. reported that in eyes with predominantly peripheral lesions, the macular vascular metrics did not correlate well with retinopathy severity [37]. Previous studies have demonstrated that in some cases the periphery of the retina is affected by ischemia, while the central retina is largely unaffected [38]. Nonperfusion in diabetic eyes is thought to exist on a spectrum from peripheral to posterior nonperfusion, with eyes of varying nonperfusion ratios in between. It is possible to say that decreased temporal–perifoveal DCP-VD predicts the presence of peripheral ischemia, but the reverse is not necessarily true. Preservation of temporal–perifoveal DCP-VD does not necessarily mean that there is no NPA or neovascularization in the peripheral regions. Therefore, changes in the temporal–perifoveal region of the DCP may be a predictor of peripheral ischemia and lead to early detection of DR progression. No significant difference was found among the groups with BCVA, but DR progression will lead to poor eyesight. It will be difficult to take widefield OCTA in patients with poor visual fixation, and we can prove that macular OCTA is useful for simple and quick determination. These OCTA quantitative vascular measurements may be used as biomarkers to detect changes with DR progression.

This study had several limitations. First, OCTA itself is subject to image artifacts and automated segmentation errors, especially when DME is present. However, there was no significant difference in central macular thickness with respect to DME, which affects vessel density measurements, and we believe that the factors that affect vascular density measurements have been eliminated as much as possible. The evaluation of vascular changes can be influenced by signal intensity. If the signal strength is low, details of the VD cannot be observed. Therefore, we used only OCTA images with a signal strength of > 6/10. We often saw poor image quality in the peripheral areas in the widefield OCTA images, widefield OCTA was standardized at 18 × 18 mm. Second, we included patients who had undergone anti-VEGF injections and/or PRP. In particular, in the PDR group, more than 90% of the patients had undergone PRP because after treatment at another hospital, patients who need further treatment are referred to a university hospital. Several studies have reported that NPA appears stable after anti-VEGF injections and/or PRP [39, 40]. Russell et al. have noted that it is difficult to determine whether the vascular dropout is a result of PRP or simply reflects the course of retinal ischemia in PDR [39]. Although retinal ischemia can progress during the course of PDR, PRP is not the cause and adjusting for PRP treatment will not change the outcome. To avoid any potential impact of the treatments, eligibility criteria was established based on these results, and only patients who had not received any injection and/or PRP in the previous 6 months were included. Further studies using OCTA are needed to investigate completely treatment-naïve retinal nonperfusion and verify whether temporal–perifoveal DCP-VD is the best predictive factor of the NPA. Additionally, we used two different OCTA machines to evaluate macular and widefield vascular parameters because RTVue has built-in software that automatically calculates vascular density, but Plex Elite does not. In addition, in order to examine whether the macular vascular density could predict the peripheral ischemia, it needed to be simple and reproducible. Therefore, vascular density was evaluated using existing software rather than Image J or other software. The standardization of the quantitative analysis of OCTA images for research and clinical practice has been suggested to be difficult because of incomparability among OCTA devices [41]. Various algorithms, wavelengths, scan patterns, image resolution, segmentation definitions, and image-processing protocols such as denoising and artifact removal produce differences in retinal vessel measurements. Further studies using the same OCTA are needed to investigate that impairment in the temporal–perifoveal DCP region is the best predictive factor for the NPA. In this study, widefield OCTA was used to assess peripheral ischemia. Widefield FA image has a wider angle of view than widefield OCTA, and widefield FA is superior in terms of wide-angle capability. However, fluorescence leakage masks ischemic areas, it will underestimate NPA. Since FA image is changing after contrast injection, it can be said that the quantification of NPA is influenced by which phase of the FA image is used. In this respect, OCTA images are the same no matter when the image is taken and the NPA quantification is accurate. Therefore, compared to previous studies, this study can objectively detect NPA and is reproducible. Finally, this study includes the relatively small number of eyes. We assume that this is because of strict criteria by excluding patients with severe diabetic complications and those who had anti-VEGF injections and/or PRP therapy within 6 months. Further study is needed to increase the number of cases in the future. However, we believe that ocular complications and low signal intensity of OCT images narrowed the number of patients initially enrolled, thus increasing the reliability of this study.

In summary, diabetic macular nonperfusion was significantly associated with the NPA of DR. In a busy outpatient setting, it is difficult to perform widefield OCTA image on all patients. Therefore, it is clinically very useful to perform macular OCTA at first, because if the temporal-perifoveal DCP has dropped out, it can be predicted that there is NPA in the peripheral as well. Our study suggests that impairment in the temporal–perifoveal DCP region from macular OCTA indicates that peripheral ischemia correlated with DR severity.
